# Bioelectrical impedance analysis to estimate body composition, and change in adiposity, in overweight and obese adolescents: comparison with dual-energy x-ray absorptiometry

**DOI:** 10.1186/1471-2431-14-249

**Published:** 2014-10-03

**Authors:** Ching S Wan, Leigh C Ward, Jocelyn Halim, Megan L Gow, Mandy Ho, Julie N Briody, Kelvin Leung, Chris T Cowell, Sarah P Garnett

**Affiliations:** School of Molecular Bioscience, University of Sydney, Sydney, Australia; School of Chemistry and Molecular Biosciences, The University of Queensland, Brisbane, Australia; Institute of Endocrinology & Diabetes, The Children’s Hospital at Westmead, Locked Bag 4001, Westmead, NSW2145 Australia; The Children’s Hospital at Westmead Clinical School, University of Sydney, Sydney, Australia; Department of Nuclear Medicine, The Children’s Hospital at Westmead, Sydney, Australia; Kids Research Institute, The Children’s Hospital at Westmead, Sydney, Australia

**Keywords:** Obese, Bioelectrical impedance analysis, Dual-energy X-ray absorptiometry, Adolescents, Cole-Cole plot

## Abstract

**Background:**

There is a need for a practical, inexpensive method to assess body composition in obese adolescents. This study aimed to 1) compare body composition parameters estimated by a stand-on, multi-frequency bioelectrical impendence (BIA) device, using a) the manufacturers’ equations, and b) published and derived equations with body composition measured by dual-energy x-ray absorptiometry (DXA) and 2) assess percentage body fat (%BF) change after a weight loss intervention.

**Methods:**

Participants were 66 obese adolescents, mean age (SD) 12.9 (2.0) years. Body composition was measured by Tanita BIA MC-180MA (Tanita BIA_8_) and DXA (GE-Lunar Prodigy). BIA resistance and reactance data at frequencies of 5, 50, 250 and 500 kHz, were used in published equations, and to generate a new prediction equation for fat-free mass (FFM) using a split-sample method. Approximately half (n = 34) of the adolescents had their body composition measured by DXA and BIA on two occasions, three to nine months apart.

**Results:**

The correlations between FFM (kg), fat mass (kg) and %BF measured by BIA and DXA were 0.92, 0.93 and 0.78, respectively. The Tanita BIA_8_ manufacturers equations significantly (P < 0.001) overestimated FFM (4.3 kg [-5.3 to 13.9]) and underestimated %BF (-5.0% [-15 to 5.0]) compared to DXA. The mean differences between BIA derived equations and DXA measured body composition parameters were small (0.4 to 2.1%), not significant, but had large limits of agreements (~ ±15% for FFM). After the intervention mean %BF loss was similar by both methods (~1.5%), but with wide limits of agreement.

**Conclusion:**

The Tanita BIA_8_ could be a valuable clinical tool to measure body composition at the group level, but is inaccurate for the individual obese adolescent.

## Background

Assessment of paediatric body composition is of increasing interest for routine monitoring of treatment efficacy, including weight loss interventions. The most commonly used measure of adiposity is body mass index (BMI), however, BMI does not differentiate between fat mass (FM) and fat-free mass (FFM), and is a poor predictor of body fat. Reference methods for determining body composition, including dual-energy x-ray absorptiometry (DXA), are costly, time consuming and frequently difficult to access. In addition, a significant number of obese individuals cannot be scanned by DXA, because they exceed the weight limitations or their body size exceeds the scanning area [1]. An alternative method is bioelectrical impedance analysis (BIA). BIA is quick, safe, non-invasive and relatively inexpensive. BIA gives estimates of total body water (TBW), determined by impedance, from which prediction models are used to estimate FFM. However, there is a great variety of BIA devices, which may be single or multi-frequency, or spectroscopic, and includes hand-to-hand, foot-to-foot and hand-to-foot systems. There is also a great variety of prediction equations, which have been recently reviewed, resulting in large, inconsistent variations in estimated body composition parameters [2].

The multi-frequency, hand-to-foot, 8-electrode BIA (BIA_8_) approach is of particular interest as it estimates whole body composition, unlike the foot-to-foot devices where the electrical current by-passes the trunk and arms. In addition, it is a stand-on device, providing greater subject convenience than electrode lead-based methods. This system has been shown to have greater accuracy in assessing DXA percentage of body fat (%BF) compared to single-frequency, 4-electrode BIA [3, 4]. We identified two previous studies which targeted overweight and obese adolescents [5, 6]. Both used a single frequency BIA_8_ system and reported underestimation of FM by the in-built manufacturers’ equations compared to DXA [5] and to a three-component model of body composition [6]. Age- and population-specific equations appear to outperform the manufacturers’ in-built equations [6]. To our knowledge, comparisons between body composition parameters, estimated by multi-frequency BIA_8,_ and a reference body composition method have not been examined in overweight and obese adolescents.

This study aimed to 1) compare body composition parameters estimated by the stand-on, multi-frequency BIA device, the Tanita BIA MC-180MA (Tanita BIA_8_), using a) the manufactures equations, and b) published and derived equations using raw data (resistance (R) and reactance (Xc)), with body composition parameters measured by DXA in overweight and obese adolescents and 2) assess change in %BF as measured by DXA and Tanita BIA_8_ after a weight loss intervention.

## Methods

### Participants

Sixty-six overweight and obese, Australian adolescents (30 boys and 36 girls), mean age 12.9 years (SD 2.0, range 10 and 18 years) were included in the study. Data were collected between May 2011 and July 2012 from adolescents participating in a randomised control trial, known as RESIST. The aim of RESIST was to examine effects of two different diets on insulin sensitivity of overweight and obese adolescents with clinical features of insulin resistance and/or prediabetes. Selection criteria and details of the RESIST study have been presented elsewhere [7]. In brief, all adolescents were overweight or obese with either pre type 2 diabetes and/or clinical features of insulin resistance. Adolescents with diabetes or secondary causes of obesity were excluded. All participants who had their body composition measured by both impedance and DXA, on the same day, were included in this study. After an overnight fast, adolescents attended an all-day appointment at The Children’s Hospital at Westmead. Participants were requested to wear light clothing (for example t-shirt and shorts) without metal; those wearing metal (for example jeans) were dressed in a hospital gown for body composition measures. On arrival the adolescents had a two hour oral glucose tolerance test after which they were offered a light lunch (sandwich and juice). Body composition was measured after lunch, in a random order depending upon availability of equipment (DXA and BIA). The maximum time difference between measures was approximately two hours. Half (n = 34; 15 female) of the adolescents had their body composition measured by DXA and BIA on two occasions, three to nine months apart. There were no statistical differences in anthropometry or body composition measures between those who had repeat measures compared to those that did not. The study was approved by The Children’s Hospital at Westmead (CHW) Human Research Ethics Committee (07/CHW/12) and written informed consent from parents and assent from the adolescents was sought prior to enrolment.

### Anthropometry

Height was measured to the nearest 0.1 cm by a calibrated stadiometer and weight was measured to the nearest 0.1 kg using standard procedures as previously described [8]. BMI was calculated as weight (kg)/height (m^2^). Overweight and obesity were defined using the International Obesity Task Force (IOTF) criteria [9]. Height, weight and BMI z-scores were calculated using the British 1990 reference data [10].

### Pubertal status

Pubertal status of the adolescents was categorized according to the Tanner Scale after assessment by the study physician. Subjects were then categorized as ‘pre-pubertal’ (Tanner 1 or 2) and ‘pubertal’ (Tanner 3 to 5).

### Bioelectrical impedance analysis

Resistance (R in ohm) and reactance (Xc in ohm) were measured with a multi-frequency (5, 50, 250 and 500 kHz) stand-on hand-to-foot 8-electrode body composition analyser, Tanita MC-180MA (Tanita, Tokyo, Japan), according to manufacturer’s instructions. Normal, non-athletic body type was chosen for the manufacture’s in-built predictive algorithm. Standard positioning was used as described in the instruction manual in all measurements and skin-to- skin contact was avoided. In brief, participants were asked to stand with bare feet on the electrode panel and hold electrodes in both hands; arms were extended and hung down in a natural standing position with the electrodes in contact with thumb and palm during the measurements. The procedure took approximately 60 seconds. The Tanita BIA_8_ measures R and Xc of both legs and arms and left side of the trunk*.* In this study, only R and Xc of the left side of the body (trunk, arm and leg combined) were used in analysis as well as FFM, FM and %BF as provided by the manufacturer’s software.

### Dual energy x-ray absorptiometry

Whole-body DXA scanning (Prodigy equipped with propriety software version 13.6, GE-Lunar, Madison, WI USA) was used as the reference body composition measurement. The manufacturer-recommended scan mode, as determined by height and weight, was used for total body mass measurements. Standard positioning techniques were used except for subjects (n = 11) who exceeded the maximum scan width. These subjects were ‘mummy wrapped’; ie the adolescent’s torso and arms are wrapped tightly in a cotton sheet. This holds the arms against the body, minimising the ‘air gaps’ between the arms and torso. Scans were analysed using manufacturer recommended techniques to provide measures of total body FFM, FM and %BF.

### Body composition prediction equations

#### Published BIA equations

The following BIA equations were used to estimate TBW or FFM:

1. Ramirez *et al*. [11]
2. Bray *et al*. [12]

where R_50_ is the resistance measured at 50 kHz (ohm), H is height (cm) and W is weight (kg). TBW was converted to FFM using a hydration fraction of 0.732 ml/g.

These equations were selected because: the outcome measures were of interest (TBW and FFM); the ages of the participants were comparable to those of the adolescents participating in the RESIST study; a large sample size of multi-ethnic, boys and girls, were included in the generation of the equations and the equations were validated against an accepted reference method (isotope dilution) [11, 12].

#### Bioimpedance spectroscopy

R and Xc of the four frequencies (5, 50, 250 and 500 kHz) provided by the Tanita BIA_8_ were used to estimate resistance at infinite frequency (R_∞_) as described by Ward *et al.* [13]. Impedance at characteristic frequency (Zc) was also determined according to the Cole model for body impedance as previously described [13]. These data were then used to predict FFM according to mixture theory using the Jaffrin equation [14].


where ρ_tbw_ is the resistivity of TBW (males, 104 ohm.cm; females, 97 ohm.cm), [15] k_b_ is a body proportion factor (3.7 calculated according to DeLorenzo et al. [16] from published anthropometric data for this age group), H is height in cm, W is weight in kg, R_∞_ is resistance at infinite frequency and D_b_ is body density (1.05 g/ml). TBW was converted to FFM using a hydration fraction of 0.732 ml/g.

#### Derived equations

To develop the prediction equations for FFM, the participants were randomly split, stratified by sex, in to two groups (Group A and B; n = 33 per group), in Excel. There were no statistical differences (P > 0.05) in the age, anthropometric or DXA body composition parameters between the groups. Equations developed in each group were cross-validated by the other group. The equations were developed by stepwise multiple regression analysis. FFM was the outcome measure and the predictor variables examined were weight, age, sex (male = 1, female = 2), pubertal stage and resistance index (height^2^/resistance or impedance at each frequency examined). Variables were entered into the equation based on the strength of the univariate association with the outcome measure and only variables with significance <0.05 were included in the final models. Frequencies examined were the Tanita BIA_8_ measured resistance at 50 kHz (R_50_) and the computed resistance, R_∞_, and impedance Zc. Age and pubertal stage were not found to be significant predictors in any of the models, consequently the weight, sex and the resistance index were the only predictors included in model development. Assumptions of normality and constant variance made in multiple regressions were checked and met. Multi-collinearity between independent variables was assessed by determining the variance inflation factor (VIF); a value <5 was considered acceptable. Covariance analysis and comparison of the slopes and intercepts were used to compare the regression models from the two groups. All equations had effectively identical predictive power as indicated by the Lin’s concordance correlation and SEE values and a single equation from the whole sample was generated.

### Statistical analysis

Statistical analysis was performed using IBM SPSS statistics 19.0 (IBM, Armonk, NY, USA) and MedCalc for Windows 13.0.0.0 (MedCalc Software, Broekstraat 52, B-9030 Mariakerke, Belgium). Sex differences were examined by independent sample t-test for continuous variables and Pearson Chi-Square test for categorical variables. Data were assessed for normality using Kolmogorov-Smirnov test and for outliers using generalized extreme studentized deviate (ESD) procedure at an alpha level of 0.05. Data for two participants were determined to be outliers. Re-examination of data for these participants failed to identify any errors in data measurement or data entry and data for these participants were retained in analyses. In addition, with 66 participants in the study, a test working at the 0.05 level would be expected to find approximately 3 (0.05 x 66) ‘outliers.’ Covariance analysis and comparison of the slopes and intercepts were used to compare the regression models between the two groups. The performance of the equations was assessed using Pearson correlation (*r*_*p*_), Lin’s concordance correlation (*r*_*c*_), and Bland-Altman limits of agreement analysis [17]. Statistical significant was set at *P* <0.05.

## Results

Consistent with our clinical population, the participants were ethnically diverse. While most (60/66) of the participants were born in Australia, only 24 reported having both parents born in Australia and/or New Zealand and of these, three had at least one parent who was an Aboriginal/Torres Strait Islander. The country of birth of the remaining parents of the participants included Southern/Central Asia (n = 9), Europe (n = 8), North Africa/Middle East (n = 7), Pacific Islands (n = 4) and South East Asia (n = 4). Anthropometric measurements and DXA body composition data are shown in Table 1. Raw anthropometric measures indicated that boys were significantly taller and heavier than girls, but there was no difference in height and weight z-scores. There was also no significant sex difference in DXA FM (kg), although boys had a significantly higher DXA FFM (kg), compared to girls, Table 1.

**Table 1:** **Anthropometry and body composition parameters determined by dual-energy X-ray absorptiometry (DXA)**

	Boys (n = 30)	Girls (n = 36)	Total (n = 66)	***P*** ^a^
Age	12.90 ± 1.95	12.86 ± 2.14	12.88 ± 2.04	0.939
Pubertal status n (%) Tanner stage ≥ 3	16 (53.3%)	26 (74.3%)	42 (64.6%)	0.078^c^
Anthropometry				
Height (cm)	165.5 ± 11.8	160.4 ± 9.1	162.8 ± 10.6	0.052
Height z-score	1.52 ± 1.57	1.21 ± 1.24	1.35 ± 1.39	0.373
Weight (kg)	95.20 ± 20.7	84.17 ± 19.4	89.2 ± 20.5	0.028
Weight z-score	3.31 ± 0.71	3.19 ± 0.91	3.24 ± 0.82	0.541
BMI	34.5 ± 5.5	32.5 ± 5.9	33.4 ± 5.8	0.149
BMI z-score	3.27 ± 0.51	2.98 ± 0.66	3.11 ± 0.61	0.057
Obese n (%)^b^	28 (93.3%)	31 (86.1%)	59 (89.4%)	0.343^c^
Overweight n (%)^b^	2 (6.7%)	5 (13.9%)	7 (10.6%)	
Reference body composition (DXA)				
Fat mass (kg)	41.79 ± 11.27	40.67 ± 12.69	41.18 ± 12.00	0.708
Fat-free mass (kg)	52.94 ± 13.26	42.81 ± 7.71	47.42 ± 11.67	0.001
Bone mineral content (kg)	2.75 ± 0.65	2.62 ± 0.67	2.67 ± 0.66	0.430
Total body fat %	45.37 ± 8.00	49.30 ± 6.63	47.51 ± 7.49	0.033

Results are presented as mean ± standard deviation unless otherwise indicated.

^a^P sex differences determined by independent sample t-test unless otherwise indicated.

^b^Overweight and obese defined by IOTF BMI criteria [9].

^c^Pearson Chi-Square test.

### Body composition parameters predicted by the in-built Tanita BIA_8_ equations and DXA

**Figure 1:**
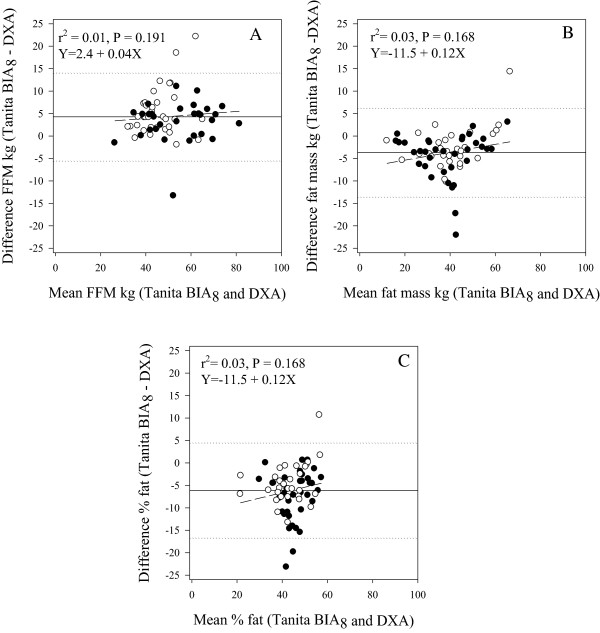
**Mean-vs-difference plots of body composition parameters determined by dual-energy x-ray absorptiometry (DXA), and in-built Tanita BIA**_**8**_ **equations (n = 66).** 1A Fat-free mass. 1B Fat mass. 1C Percentage of body fat. Key ○ Boys ● Girls. ……. Limits of agreement (±1.96 SD) (dotted). ―Bias (solid). ----Line of best fit (short dash).

Figure 1 compares FFM, FM and %BF predicted by the in-built Tanita BIA_8_ equations and measured by DXA. The correlations (*r*_*p*_) between measures were 0.92, 0.93 and 0.78 for FFM, FM and %BF, respectively. However, the strength of agreement between pairs of measures was poor; concordance correlations (*r*_*c*_) for FFM, FM and %BF were 0.86, 0.87 and 0.65 respectively. The manufacturers’, in-built Tanita BIA_8_ equations significantly (P < 0.001) overestimated FFM (mean difference 4.3 kg) and underestimated FM and %BF (mean difference 5.0%) compared to DXA, with large 95% limits of agreement, for example -15.1 to 5.0 for %BF.

### Body composition predicted using published equations, based on the resistance and reactance data from the Tanita BIA_8_ and DXA

The comparison between body composition parameters calculated using the Ramirez et al. [11] and Bray et al. [12] equations, based on resistance, as well as body composition parameters calculated using the Jaffrin et al. equation [14], based on resistance and reactance data from the Tanita BIA_8_, and DXA are shown in Table 2. Predictions of FFM and FM from all the equations were highly correlated with DXA FFM and FM, *r*_*p*_ = 0.93 to 0.95; P < 0.001. The correlation with %BF was weaker; *r*_*p*_ = 0.81 to 0.82; P < 0.001. The strength of agreement between pairs of measures was moderate to substantial for all equations predicting FFM and FM compared to DXA measures; concordance correlations, *r*_*c*_ = 0.93 to 0.95, respectively. Poor concordance correlations were observed with all three predictions of %BF, *r*_*c*_ = 0.76 to 0.79, Table 2.

**Table 2:** **Body composition parameters determined by dual-energy x-ray absorptiomtry, in-built Tanita BIA**_**8**_ **equations, and published equations**

Method	DXA	Tanita BIA _8_	Ramirez et al. [ [11]]	Bray et al. [ [12]]	Jaffrin et al. [ [14]]
Fat-free mass kg					
Mean ± SD (kg)	47.4 ± 11.7	51.7 ± 12.1^a^	48.0 ± 11.1	47.8 ± 9.8	48.3 ± 11.5
Bias (kg)		4.3	0.6	0.4	0.9
Limits of agreement (kg)		-5.3 to 13.9	-7.1 to 8.2	-7.6 to 8.4	-6.2 to 8.0
*r*_*c*_ (95% CI)^b^		0.86 (0.79 – 0.91)	0.94 (0.90 – 0.96)	0.93 (0.89– 0.6)	0.95 (0.92 – 0.97)
Fat mass kg					
Mean ± SD (kg)	41.8 ± 12.1	37.5 ± 13.0^a^	41.2 ± 11.5	41.3 ± 12.2	40.9 ± 11.6
Bias (kg)		-4.3	-0.6	-0.5	-0.9
Limits of agreement (kg)		-13.9 to 5.3	-8.2 to 7.1	8.4 to -7.6	8.0 to -6.2
*r*_*c*_ (95% CI)^b^		0.87 (0.81 – 0.92)	0.94 (0.91 – 0.96)	0.94 (0.91 – 0.96)	0.95 (0.92 – 0.97)
Percentage of body fat					
Mean ± SD (%)	46.4 ± 7.4	41.4 ± 8.3^a^	45.8 ± 5.5	45.7 ± 5.2	45.6 ± 6.0
Bias (%)		-5.0	-0.6	-0.5	-0.8
Limits of agreement (%)		-15.1 to 5.0	-9.1 to 7.9	-9.2 to 8.0	-9.2 to 7.4
*r*_*c*_ (95% CI)^b^	-	0.65 (0.53 – 0.75)	0.77 (0.67 - 0.85)	0.76 (0.65 – 0.83)	0.79 (0.69 – 0.86)

^a^difference between DXA and Tanita BIA_8_, P <0.001.

^b^CI: confidence interval.

All three predictive methods overestimated FFM and underestimated FM and %BF compared to DXA measurements. The mean differences were small (0.2 to 0.5 kg) and were not statistically significant. Nor was there any statistically significant difference between the three methods for FFM, FM or %BF. However, the limits of agreement for all equations were large; approximately ± 8 kg (±15% of DXA measurement) for FFM, ± 8 kg (±20% of DXA measurement) for FM and ±8% (±15% of DXA measurement) %BF, Table 2. Examination of the differences-vs-means plots (data not shown) for each predictive model for FFM indicated that the Bray et al. prediction equation of DXA FFM, had a significant positive slope (FFM_DXA_ = -8.75 + 0.18FFM_Bray;_ P < 0.001), despite exhibiting a similar bias and limits of agreement to the other two equations.

### Fat-free mass predicted using derived equations, based on the resistance and reactance data from the Tanita BIA_8_ and DXA

The derived regression models for prediction of FFM using sex, weight and with and without different resistance indices are shown in Table 3. All models which included a resistance index had SEE similar in magnitude (3.57 to 4.23 kg FFM) and there was no statistical significant differences between groups for any of the regression models. The proportion of the variance explained by the independent variables was high for all models (*r*^2^ = 0.86 to 0.93). Standardised partial regression coefficients for each independent variable were also similar between groups for each of the models with the resistance indices explaining approximately 60% and weight 35% of the variance. In all cases, sex accounted for less than 10% of the variance in the models.

**Table 3:** **Prediction equations for fat-free mass based on different resistance indices (RI)**

Group		Regression coefficients						
	n	RI	Sex	Weight	Constant	***r*** ^2^	SEE ^a^	***P***
*Resistance index*			*nil*					
All subjects	66	-	-5.114	0.454	14.867	0.773	5.56	0.001
Resistance index			*H* ^*2*^ */R*_*50*_					
A	33	0.615	-2.906	0.213	4.294	0.918	3.79	0.001
B	33	0.578	-2.589	0.204	6.777	0.881	3.93	0.001
All subjects	66	0.589	-2.849	0.213	5.657	0.901	3.76	0.001
*Resistance index*			*H* ^*2*^ */R*_*∞*_					
A	33	0.393	-3.062	0.259	4.645	0.907	4.03	0.001
B	33	0.481	-2.453	0.173	6.392	0.902	3.57	0.001
All subjects	66	0.444	-3.001	0.212	5.846	0.902	3.73	0.001
*Resistance index*			*H* ^*2*^ */Zc*					
A	33	0.718	-2.202	0.195	0.156	0.926	3.60	0.001
B	33	0.561	-3.240	0.219	7.104	0.863	4.23	0.001
All subjects	66	0.612	-2.936	0.217	4.354	0.896	3.85	0.001

RI examined were height^2^/resistance 50 kHz (H^2^/R_50_), height^2^/estimated resistance at infinity (H^2^/R_∞_) and height^2^/impedance at characteristic frequency (H^2^/Zc).

^a^SEE standard error of estimate.

There were no significant differences between the estimates of FFM from derived equations, based on different resistance indices and DXA FFM for any of the models, Table 4. The lack of difference may have been anticipated as the derived equations were based on the DXA data. The Pearson’s correlation coefficients and concordance coefficients were identical for each model and varied between 0.93 and 0.95. Similar to the previously published equations of Ramirez et al. and Bray et al. (Table 2) the mean estimates were 0.5 kg (~1%) of DXA FFM. Limits of agreement were similar, for all models approximately ± 7 kg (±15%). There were no statistically significant variations in bias across the range of FFM.

**Table 4:** **Fat-free mass determined by dual-energy x-ray absorptiometry and derived equations based on different resistance indices (RI)**

Method	Group A (n = 33)	Group B (n = 33)	P
DXA Mean ± SD (kg)	47.9 ± 12.6	46.9 ± 10.9	0.682
H^2^/R_50_			
Mean ± SD (kg)	48.3 ± 11.4	46.5 ± 10.8	0.519
Bias (kg)	0.4	-0.5	
Limits of agreement (kg)	-6.8 to 7.6	-7.9 to 7.0	
*r*_*c*_ (95% CI)	0.95 (0.91 – 0.97)	0.94 (0.88 – 0.97)	
H^2^/R_∞_			
Mean ± SD (kg)	48.3 ± 11.4	46.5 ± 10.6	0.516
Bias (kg)	0.4	-0.4	
Limits of agreement (kg)	-7.5 to 8.2	-7.5 to 6.6	
*r*_*c*_ (95% CI)	0.94 (0.89 – 0.97)	0.94 (0.89 – 0.97)	
H^2^/Zc			
Mean ± SD (kg)	48.1 ± 11.3	46.7 ± 11.2	0.612
Bias (kg)	0.2	-0.2	
Limits of agreement (kg)	-6.9 to 7.2	-8.6 to 8.1	
*r*_*c*_ (95% CI)	0.95 (0.91 – 0.98)	0.93 (0.85 – 0.96)	

RI examined were height^2^/resistance 50 kHz (H^2^/R_50_), height^2^/estimated resistance at infinity (H^2^/R_∞_) and height^2^/impedance at characteristic frequency (H^2^/Zc).

### Change in percentage of body fat

**Figure 2:**
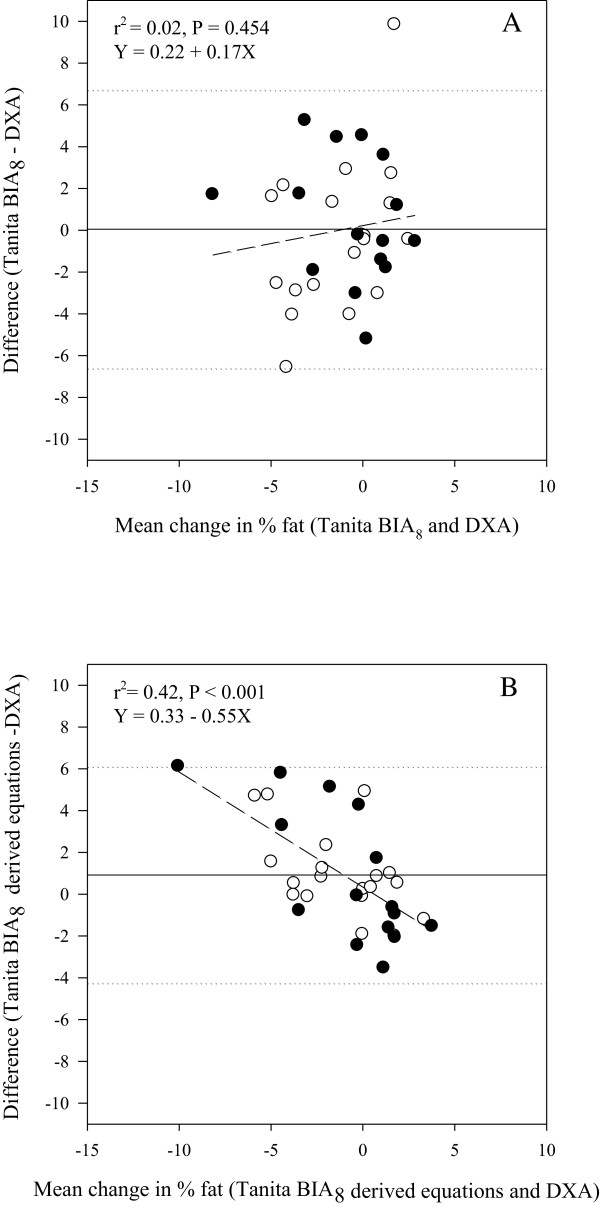
**Mean-vs-difference plots of change in percent body fat (%fat) determined by dual-energy x-ray absorptiometry (DXA), compared to A) in-built Tanita BIA**_**8**_ **equations and B) derived equation using the resistance index height** ^**2**^ **/R**_**50**_ **(n = 34).** ○ Boys ● Girls. ……. Limits of agreement (±1.96 SD) (dotted). ―Bias (solid). ----Line of best fit (short dash).

The mean %BF loss measured of the 34 adolescents that had body composition measured on two occasions by DXA was -1.5% ± 4.0 and did not differ (-1.5% ± 4.4, P = 0.933) from that determined by the in-built Tanita BIA_8_ equations, albeit with wide limits of agreement, Figure 2a. The estimated %BF change derived from the equation based on RI H^2^/R_50_ was similar, -0.6% ± 2.4, but statistically different compared to the other estimates (P < 0.05), and showed significant bias; a strong association was observed whereby the loss of %BF was overestimated and gain in %BF was underestimated, Figure 2B. The correlation (*r*_*p*_) between change in %BF as measured by DXA was 0.69 and 0.78 for in-built Tanita BIA_8_ equations and the derived equation based on RI H^2^/R_50_, respectively. However, the strength of agreement between pairs of measures was poor; concordance correlations, *r*_*c*_ = 0.69 and 0.66.

## Discussion

In this ethnically diverse, overweight and obese adolescent population, there are strong correlations between FFM, FM and %BF measured by Tanita BIA_8_ and DXA. However, the manufacturers’ ín-built equations significantly overestimated FFM and underestimated FM by ~4 kg (9 to 10%), with wide limits of agreement (~19 kg), compared to DXA measurements. By using the Tanita BIA_8_ resistance data with published [11, 12] and our own derived equations the bias was reduced to a clinically acceptable level of <1.0 kg (<2%), but the limits of agreement remained wide (~15 kg;). These results indicate that using derived equations, Tanita BIA_8_ is potentially useful for measuring body composition in overweight and obese adolescent populations, but is inaccurate for the individual.

The over estimation of FFM by the manufacturers’ equations and improved agreement with derived equations, are consistent with the two other studies that have examined the relation between BIA_8_ single frequency system (Tanita BC-418MA) and DXA [5] and a three-component model of body composition, [6] in overweight and obese adolescents, with a white ethnic background. The results are also broadly consistent with other paediatric studies which have compared estimates of body composition measures by BIA_8_ (differing in manufactures/models) with reference body composition methods in healthy children, of various ages, nutritional status and ethnicity including Korean [18] rural Gambian [19], New Zealand European, Pacific islander, Asian and Maori [20]. However, recent evidence indicates that standardising BIA measurements, using new paediatric body composition reference data, [1] could be a reasonable measure of four compartment FFM if DXA was not available [21] and is worthy of further research. In the absence of an independent cohort our derived models were based on DXA body composition parameters and cross validated [2]; this may explain the improved agreement from the derived models with DXA, compared with the BIA_8_ manufacturers’ estimates.

The mean change in %BF over time was low (-1.5%) and maybe within the error made by DXA for repeated measures. Nevertheless, a strong correlation was also observed between change in %BF as measured by DXA and Tanita BIA_8_ (manufactures’ and derived equations) and the estimated mean change in %BF over time, was similar. However, both measures, compared to DXA had large limits of agreements in %BF change. Change in %BF estimated by Tanita BIA_8_ using derived equations also showed significant bias whereby the loss of %BF was overestimated and gain in %BF was underestimated, Figure 2B. This bias was not observed using the manufacturers’ equations. The Tanita BIA_8_ in-built equations could be used to measure overall change in a group of overweight and obese adolescents, but not for an individual.

Consistent with other studies [22], all three of our derived equations using different resistance indices (height^2^/resistance 50 kHz (H^2^/R_50_), height^2^/estimated resistance at infinity (H^2^/R_∞_) and height^2^/impedance at characteristic frequency (H^2^/Zc) included sex and weight and explained a high proportion of the variability in FFM (86 to 96%). It was interesting to note that there were no significant differences between the estimates of FFM using the different indices; that is, the equation using the 50 kHz single frequency performed as well as the equation using the multi frequency resistance indices. This, while not tested in this study, indicates that if BIA is to be used to measure total FFM and FM, the cheaper single frequency models may be adequate.

Our study had a number of limitations, including using DXA as a reference method. DXA relative to the four-compartment model of body composition had been reported to overestimate adiposity by more than 20% in obese individuals [23, 24]. Given this uncertainty in the reference method, BIA_8_ might represent the ‘true’ average value for adiposity in this population, however, further work is required to clarify this issue. In addition, both DXA and Tanita BIA_8_ assume a constant hydration factor of FFM which is known to change during childhood with age [2]; adiposity estimated by BIA_8_ and DXA should be interpreted with caution. The composition of FFM is also reported to be significantly different in obese compared to lean children and may vary between moderately and extremely obese children [25]. In the obese the water and mineral content are higher such that the proportion of protein is reduced; hence the hydration of FFM is reported to be significantly higher in obese children (79.2%) compared to than leaner children (76.7%) [12]. However the differences in hydration of FFM may have only a small effect on %BF (<0.3%) [12]. The important advantage that BIA has compared to DXA is the ability to measure the severely obese individuals, who are too heavy or wide to be measured by DXA, leading to exclusion from clinical research studies from which the obese individuals may benefit. A recent body compositions study indicated that this could be >13% of children and adolescents [24]. Another study limitation was that the adolescents were not fasted when body composition was measured. Consumption of food and beverages has been reported to decrease impedance However, the errors are considered small (<3%) [26].

Some previous studies [27, 28], but not all [11, 29] have shown ethnic variability between resistance indices and body composition in adolescents. Due to the heterogeneity of our study population it was not possible to explore this association. Pubertal stage in some studies has also been shown to alter the relation between FFM and resistance indices [30]. Puberty was tested in the Tanita BIA_8_ derived equations but was not a significant predictor. It is not clear if this is a real finding or due to the limited age range, the degree of adiposity of our study population and/or the study was underpowered to identify the differences.

## Conclusions

In conclusion, there is an increasing need in both the clinical and research setting for a practical, accurate and inexpensive method to assess adiposity in overweight and obese children and adolescents. BMI and DXA, have significant limitations. BMI will fail to demonstrate improved body composition if the proportion of FFM to FM changes, for example after a physical activity program and an increasing number of obese individuals cannot be scanned by DXA because of their weight and body width. BIA is a rapid, safe and non-invasive method of measuring body composition with relatively good ranking consistency of FFM and FM and could be a valuable clinical tool at the group level.

## Abbreviations

BIA: Bioelectrical impendence analysis
DXA: Dual energy x-ray absorptiometry
Tanita BIA_8_: Tanita BIA MC-180MA
FFM: Fat-free mass
FM: Fat Mass
%BF: Percentage body fat
BMI: Body Mass Index
R: Resistance
Xc: Reactance.
